# Saccharification of rice straw by cellulase from a local *Trichoderma harzianum* SNRS3 for biobutanol production

**DOI:** 10.1186/s12896-014-0103-y

**Published:** 2014-12-12

**Authors:** Nooshin Rahnama, Hooi Ling Foo, Nor Aini Abdul Rahman, Arbakariya Ariff, Umi Kalsom Md Shah

**Affiliations:** Department of Bioprocess Technology, Faculty of Biotechnology and Biomolecular Sciences, Universiti Putra Malaysia, 43400 UPM Serdang, Selangor Malaysia; Institute of Bioscience, Universiti Putra Malaysia, 43400 UPM Serdang, Selangor Malaysia; Institute of Tropical Forestry and Forest Products, Universiti Putra Malaysia, 43400 UPM Serdang, Selangor Malaysia; Bioprocessing and Biomanufacturing Research Center, Faculty of Biotechnology and Biomolecular Sciences, Universiti Putra Malaysia, 43400 UPM, Serdang, Selangor Malaysia

**Keywords:** Rice straw, Saccharification, Biobutanol, *Trichoderma harzianum* SNRS3

## Abstract

**Background:**

Rice straw has shown to be a promising agricultural by-product in the bioconversion of biomass to value-added products. Hydrolysis of cellulose, a main constituent of lignocellulosic biomass, is a requirement for fermentable sugar production and its subsequent bioconversion to biofuels such as biobutanol. The high cost of commercial enzymes is a major impediment to the industrial application of cellulases. Therefore, the use of local microbial enzymes has been suggested. *Trichoderma harzianum* strains are potential CMCase and β-glucosidase producers. However, few researches have been reported on cellulase production by *T. harzianum* and the subsequent use of the crude cellulase for cellulose enzymatic hydrolysis. For cellulose hydrolysis to be efficiently performed, the presence of the whole set of cellulase components including exoglucanase, endoglucanase, and β-glucosidase at a considerable concentration is required. Biomass recalcitrance is also a bottleneck in the bioconversion of agricultural residues to value-added products. An effective pretreatment could be of central significance in the bioconversion of biomass to biofuels.

**Results:**

Rice straw pretreated using various concentrations of NaOH was subjected to enzymatic hydrolysis. The saccharification of rice straw pretreated with 2% (w/v) NaOH using crude cellulase from local *T. harzianum* SNRS3 resulted in the production of 29.87 g/L reducing sugar and a yield of 0.6 g/g substrate. The use of rice straw hydrolysate as carbon source for biobutanol fermentation by *Clostridium acetobutylicum* ATCC 824 resulted in an ABE yield, ABE productivity, and biobutanol yield of 0.27 g/g glucose, 0.04 g/L/h and 0.16 g/g glucose, respectively. As a potential β-glucosidase producer, *T. harzianum* SNRS3 used in this study was able to produce β-glucosidase at the activity of 173.71 U/g substrate. However, for cellulose hydrolysis to be efficient, Filter Paper Activity at a considerable concentration is also required to initiate the hydrolytic reaction. According to the results of our study, FPase is a major component of cellulose hydrolytic enzyme complex system and the reducing sugar rate-limiting enzyme.

**Conclusion:**

Our study revealed that rice straw hydrolysate served as a potential substrate for biobutanol production and FPase is a rate-limiting enzyme in saccharification.

## Background

Lignocellulosic substrates are the most abundant, low cost renewable resources worldwide [1]. Cellulose, hemicelluloses and lignin are the three main components which make up lignocellulosic biomass. Via enzymatic hydrolysis, polysaccharides in the lignocellulosic substrates can be hydrolyzed to monosaccharides [2]. Saccharification is therefore a crucial step for sugar production [1]. Acid hydrolysis and enzymatic hydrolysis are the two commonly used methods for hydrolysis of cellulosic materials. Enzymatic hydrolysis is advantageous over acid hydrolysis. It has low environmental impact and the reaction is carried out under mild conditions [3]. Recently, due to the constant increase in the oil price, the significance of biofuel production from lignocellulosic biomass as an alternative energy source has been intensified and efficient conversion of lignocelluloses to biofuel is gaining interest [4].

Rice is a major crop produced in large quantities in the world [5] and rice straw is the most abundant agricultural residue worldwide. Approximately 700-800 million tons of rice straw is produced annually, most of which is found in Asia. Hence, rice straw is a suitable feedstock for biofuel production especially in Asia [2]. Various agricultural wastes have been used as the substrate for biobutanol production. However, not much research has focused on biobutanol production from rice straw as the feedstock for fermentation and limited research has been reported [6-9].

As fermentable sugar production from agricultural waste is a prerequisite for bioenergy production, enormous research attempt has focused on bioconversion of cellulose into fermentable sugar [10]. Yet, more research is required to improve the process. Hence, this work focused on fermentable sugar production from alkali pretreated rice straw by using cellulase produced from a local *T. harzianum* SNRS3 under solid state cultivation. Rice straw hydrolysate was subsequently utilized as substrate for biobutanol production.

## Results and discussion

### Effect of alkali pretreatment of rice straw on rice straw chemical composition

Alkali pretreatment of rice straw using 2% (w/v) NaOH showed a rise in cellulose content of rice straw, whereas the content of lignin and hemicelluloses was decreased. Table 1 demonstrates cellulose, hemicellulose, lignin, and ash content of rice straw both in untreated and alkali-pretreated rice straw. As shown in the table, as a result of pretreatment, rice straw was delignified and a decrease in lignin content from 9.22% to 3.78% was observed. There was a decrease in hemicelluloses content from 26.03% to 17.63% after rice straw was pretreated as well. However, cellulose content of rice straw was promoted from 39.74% in untreated rice straw to 70.9% after rice straw was subjected to alkali pretreatment.

**Table 1:** **Chemical composition of rice straw before and after alkali pretreatment**

**Pretreatment**	*Cellulose (%)	*Hemicelluloses (%)	*Lignin (%)	*Ash (%)
Untreated	39.74 ± 3.69	26.03 ± 0.30	9.22 ± 3.01	12.48 ± 0.38
2% (w/v) NaOH	70.9 ± 0.81	17.63 ± 3.07	3.78 ± 0.95	1.55 ± 0.05

*Results are based on the mass of dry matter.

In a study, [2% (w/v) NaOH] was used to pretreat rice straw and a decrease in lignin and hemicelluloses content of rice straw was reported from 15.8% and 25.8% to 9.8% and 18%, respectively, and an increase in cellulose content of alkali-pretreated rice straw from 36.8% to 52.2% was noted as well [1]. As reported in another research, chemical composition of rice straw was different before and after pretreatment by [2% (w/v) NaOH], and a decrease in lignin and hemicelluloses content of rice straw after alkali pretreatment was observed. Whereas, an increase in cellulose content occurred when rice straw was pretreated. As a result of alkali pretreatment of rice straw with [2% (w/v) NaOH], cellulose content was increased from 38.3% to 59.3% in alkali-pretreated rice straw and lignin and hemicelluloses content was decreased from 14.9% and 28% in untreated rice straw to 9.5% and 10.9% in pretreated rice straw, respectively [11].

### Effect of different concentrations of NaOH for pretreatment of rice straw on reducing sugar production

**Figure 1:**
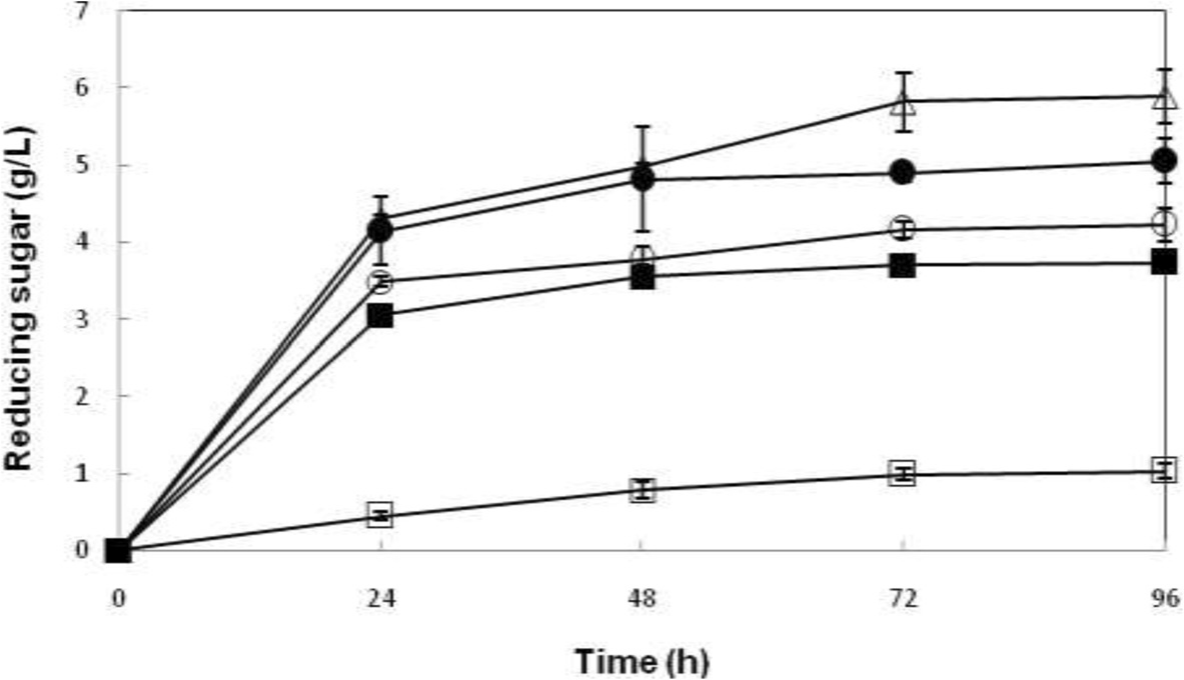
**Enzymatic hydrolysis of rice straw pretreated with different concentrations of NaOH.** Values are means of 3 replicates ± SD. Symbols represent: □: Untreated; ●: 1%; ∆: 2%; ○: 3%; ■: 4%.

Rice straw pretreated with various concentrations of NaOH [1%, 2%, 3%, and 4% (w/v)] was used as saccharification substrate and enzymatic hydrolysis of rice straw was performed. Figure 1 illustrates the reducing sugar production profile from rice straw pretreated by using different concentrations of NaOH. The reducing sugar production was increased significantly *(p* < 0.05) when NaOH-pretreated rice straw was used as substrate in enzymatic hydrolysis. As for the effect of incubation time, there was significant difference (*p* < 0.05) for the production of reducing sugar from 0-72 h from NaOH-pretreated rice straw used in enzymatic hydrolysis, except for 1% (w/v) NaOH, where the production of reducing sugar was only increased significantly from 0-48 h. The detail of the statistical analysis is presented in Table 2.

**Table 2:** **Reducing sugar production (g/L) by crude cellulase from** ***T. harzianum*** **SNRS3 from rice straw pretreated with different concentrations of NaOH**

		**NaOH Concentration (w/v)**			
**Time (h)**	**Untreated**	**1%**	**2%**	**3%**	**4%**
0	0.00^Aa^	0.00^Aa^	0.00^Aa^	0.00^Aa^	0.00^Aa^
24	0.44^Ba^	4.15^Bb^	4.31^Bb^	3.49^Bc^	3.05^Bd^
48	0.78^Ca^	4.82^Cb^	4.98^Cb^	3.78^Cc^	3.55^Cc^
72	0.99^Da^	4.90^Cb^	5.82^Dc^	4.17^Dd^	3.70^De^
96	1.03^Da^	5.06^Cb,c^	5.89^Db^	4.23^Dc^	3.73^Dd^

Note: Means expressed with different superscript capital letters within the same column was significantly different at *p* < 0.05.

Means expressed with different superscript small letters within the same row was significantly different at *p* < 0.05.

According to the results obtained in this study, the enzymatic hydrolysis of rice straw pretreated by using 2% (w/v) NaOH resulted in the highest production of reducing sugars at 5.82 g/L at 72 h of incubation, as compared to 0.99 g/L reducing sugars produced from untreated rice straw.

In a study, the effect of different concentrations of NaOH on enzymatic hydrolysis of bagasse using cellulases of *Bacillus subtilis* was investigated. As reported, the enzymatic hydrolysis of the substrate pretreated with 2% (w/v) NaOH resulted in higher production of reducing sugars, as compared to enzymatic hydrolysis of the substrate pretreated with [1%, 3%, and 4% (w/v)] NaOH. Low yield of reducing sugar obtained at [3% and 4% (w/v)] NaOH concentration could be due to the loss of carbohydrates that might have been solubilized while the substrate was being pretreated. In addition, the low saccharification could be attributed to the inaccessible insoluble cellulose. This might contribute to low sugar yield when saccharification substrates were pretreated at higher concentrations of NaOH [12].

As reported in our previous research [13], after rice straw was pretreated by using alkali, not only chemical composition of rice straw was changed, but also changes in the cellulose morphological structure occurred. The results of X-ray diffraction analysis revealed an increase in relative crystallinity of cellulose in alkali-pretreated rice straw, as compared to untreated rice straw. Hydrolyzation and peeling of amorphous regions during pretreatment could result in the rise in relative crystallinity of cellulose in alkali-pretreated rice straw. However, alkali pretreatment decreased the absolute crystallinity of cellulose. As the SEM images showed, the pretreatment process disrupted the hemicellulose and lignin, which might have resulted in the changes in the structure of cellulose. According to the results of FTIR analysis, alkali pretreatment caused lignin removal and the changes in cellulose structure.

### Effect of enzyme loadings on reducing sugar production

**Figure 2:**
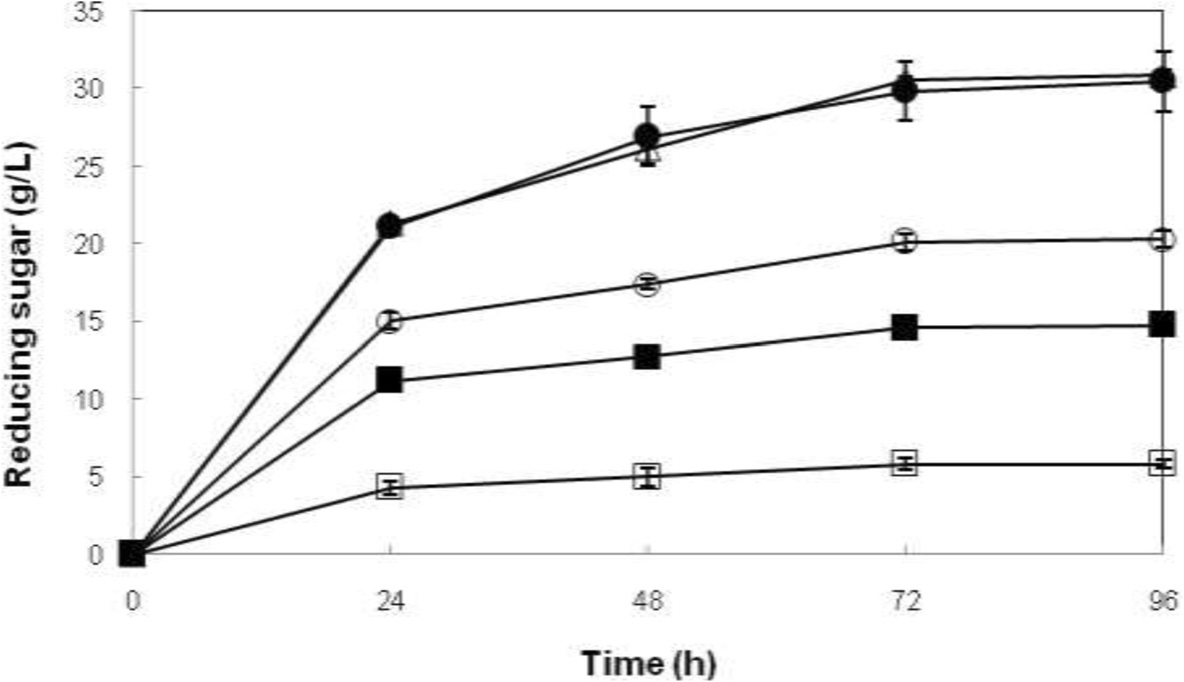
**Effect of enzyme concentration on hydrolysis of rice straw by cellulases from** ***T. harzianum*** **SNRS3.** Values are means of 3 replicates ± SD. Symbols represent enzyme activity (U/g substrate): □: FPase 6.25, CMCase 111.31, β-glucosidase 173.71; ■: FPase 31.25, CMCase 566.55, β-glucosidase 868.55; ○: FPase 62.5, CMCase 1113.1, β-glucosidase 1737.1; ●: FPase 93.75, CMCase 1669.65, β-glucosidase 2605.65; ∆: FPase 125, CMCase 2226.2, β-glucosidase 3474.2.

Enzyme loading is an important parameter in enzymatic hydrolysis, affecting reducing sugar yield. Therefore, the effect of enzyme concentration on enzymatic hydrolysis of rice straw pretreated with 2% (w/v) NaOH was investigated. Various enzyme loadings were used for hydrolysis of 5% (w/v) rice straw pretreated with 2% (w/v) NaOH as saccharification substrate. The reducing sugar production profile was studied over a period of 96 h. Figure 2 shows that the reducing sugar production was increased significantly (*p* < 0.05) according to the cellulases concentration used in enzymatic hydrolysis, except for the cellulase concentration of FPase: 125 U/g; CMCase: 2226.2 U/g; β-glucosidase: 3474.2 U/g and FPase: 93.75 U/g CMCase: 1669.65 U/g; β-glucosidase: 2605.65 U/g. As for the effect of incubation time there was significant difference (*p* < 0.05) for the production of reducing sugar from 0-72 h for all cellulase concentrations used in enzymatic hydrolysis. The concentration of crude cellulase at the activity of FPase: 93.75 U/g CMCase: 1669.65 U/g; β-glucosidase: 2605.65 U/g that resulted in the production of 29.87 g/L reducing sugar was selected for the subsequent study since the reducing sugar production was not significantly (*p* > 0.05) different from the cellulase at the activity of FPase: 125 U/g; CMCase: 2226.2 U/g; β-glucosidase: 3474.2 U/g. The detail of the statistical analysis is presented in Table 3.

**Table 3:** **Reducing sugar production (g/L) from rice straw using various concentrations of crude cellulase from** ***T. harzianum*** **SNRS3**

	**Enzyme Concentration (U/g substrate)**				
**Time (h)**	*1	*2	*3	*4	*5
0	0.00^Aa^	0.00^Aa^	0.00^Aa^	0.00^Aa^	0.00^Aa^
24	4.31^Ba^	11.19^Bb^	15.05^Bc^	21.14^Bd^	21.34^Bd^
48	4.98^Ca^	12.78^Cb^	17.40^Cc^	26.93^Cd^	26.20^Cd^
72	5.82^Da^	14.57^Db^	20.14^Dc^	29.87^Dd^	30.55^Dd^
96	5.84^Da^	14.77^Db^	20.31^Dc^	30.47^Dd^	30.87^Dd^

Note: *1: FPase 6.25 U/g, CMCase 111.31 U/g, β-glucosidase 173.71 U/g.

*2: FPase 31.25 U/g, CMCase 566.55 U/g, β-glucosidase 868.55 U/g.

*3: FPase 62.5 U/g, CMCase 1113.1 U/g, β-glucosidase 1737.1 U/g.

*4: FPase 93.75 U/g, CMCase 1669.65 U/g, β-glucosidase 2605.65 U/g.

*5: FPase 125 U/g, CMCase 2226.2 U/g, β-glucosidase 3474.2 U/g.

Means expressed with different superscript capital letters within the same column was significantly different at *p* < 0.05.

Means expressed with different superscript small letters within the same row was significantly different at *p* < 0.05.

The cost of cellulase enzymes contributes to the total cost of saccharification process. Hence, it is suggested to minimize enzyme dosage as much as possible [14]. An increase in enzyme concentration resulted in significant rise of hydrolysis rate of rice straw, wheat straw, and bagasse. However, further increase in enzyme concentration did not result in significant rise of hydrolysis rate. This might be due to improper mixing and suspension of the slurry. In fact, the rise in enzyme concentration should increase hydrolysis rate. However, it makes the process uneconomical [12].

In this study, a similar trend was noted for fermentable sugar production and conversion yield percentage. A comparison between fermentable sugar production and hydrolysis yield (%) is illustrated in Table 4.

**Table 4:** **Fermentable sugars production and hydrolysis yield (%) obtained by using various concentrations of crude cellulases from** ***T. harzianum*** **SNRS3**

**Cellulase of** ***T. harzianum*** **SNRS3(U/g)**			**Fermentable sugar (g/L)**	**Conversion/ Hydrolysis yield (%)**
FPase	CMCase	β-glucosidase		
6.25	111.31	173.71	5.82 ± 0.37 ^a^	11.83
31.25	566.55	868.55	14.57 ± 0.47 ^b^	29.63
62.50	1113.10	1737.10	20.14 ± 0.53 ^c^	40.96
93.75	1669.65	2605.65	29.87 ± 1.87 ^d^	60.75
125	2226.20	3474.20	30.55 ± 0.23 ^d^	62.13

Note: Means expressed with different superscript letters within the same column was significantly different at (*p* < 0.05).

The increase in enzyme loading favoured enzymatic hydrolysis of corncob residue [12], wheat straw, rice straw, and bagasse [12]. A similar trend was also noted for fermentable sugar production and conversion yield percentage with the increase in concentration of the enzyme used [15]. As reported in other studies, a rise in reducing sugar yield occurred as the enzyme concentration increased [12,15]. Therefore, the results of this research are in good agreement with other studies. The optimal ratio between enzyme and substrate is an important factor affecting efficiency of enzymatic hydrolysis. In fact, more active sites of the enzyme would be involved in conversion of the substrate into reducing sugars via enzymatic hydrolysis when enzyme concentration increases [5].

### Effect of substrate concentration on reducing sugar production

**Figure 3:**
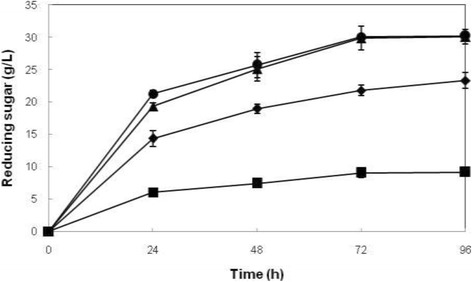
**Enzymatic hydrolysis of rice straw using different concentrations of the substrate (w/v).** Values are means of 3 replicates ± SD. Symbols represent different concentrations of the substrate (w/v): ■: 1%; ♦: 3%; ▲: 5%; ●: 7%.

Efficiency of enzymatic hydrolysis is determined by the optimal ratio between enzyme and substrate. Hence, the effect of concentration of the substrate on reducing sugar production was investigated by using different concentrations of substrate [1%, 3%, 5%, and 7% (w/v)] and the optimal unit of enzyme activity (FPase: 93.75 U/g, CMCase: 1669.65 U/g, β-glucosidase: 2605.65 U/g) based on the results obtained in the previous experiment. The reducing sugar profile was studied over a period of 96 h. Figure 3 shows the reducing sugar production was increased significantly (*p* < 0.05) according to the substrate concentration used in enzymatic hydrolysis, except for the substrate concentration at 5% and 7% (w/v). As for the effect of incubation time there was significant difference (*p* < 0.05) for the production of reducing sugar from 0-72 h for all substrate concentrations used in enzymatic hydrolysis. 5% (w/v) substrate that resulted in the production of 29.87 g/L reducing sugars was selected as substrate concentration for reducing sugar production in subsequent study since the reducing sugar production was not significantly different (*p* > 0.05) from 7% (w/v) substrate concentration. The detail of the statistical analysis is presented in Table 5.

**Table 5:** **Reducing sugar production (g/L) by crude cellulase from** ***T. harzianum*** **SNRS3 using various concentrations of rice straw as substrate**

**Time (h)**	**Substrate Concentration (w/v)**			
	**1%**	**3%**	**5%**	**7%**
0	0.00^Aa^	0.00^Aa^	0.00^Aa^	0.00^Aa^
24	6.02^Ba^	14.33^Bb^	19.30^Bc^	21.85^Bd^
48	7.39^Ca^	18.94^Cb^	25.10^Cc^	25.70^Cc^
72	9.03^Da^	21.79^Db^	29.87^Dc^	30.07^Dc^
96	9.15^Da^	23.30^Eb^	30.70^Dc^	30.23^Dc^

Note: Means expressed with different superscript capital letters within the same column was significantly different at *p* < 0.05.

Means expressed with different superscript small letters within the same row was significantly different at *p* < 0.05.

Enzymatic hydrolysis of 10% (w/v) rice straw resulted in the production of higher reducing sugar, as compared to [4% and 6% (w/v)] substrates. As a result, 18.5 g/L reducing sugar was obtained [12]. Effect of substrate concentration on reducing sugar production was investigated by using various lignocellulosic substrates such as corncob, sunflower stalks, wheat straw, rice straw, and bagasse and it was shown that the increase in substrate concentration favoured reducing sugar production [12,15,16]. In this study, an opposite trend was noted between reducing sugar concentration and hydrolysis yield (%). With the rise in substrate concentration, sugar production increased. However, hydrolysis yield percentage decreased due to end product inhibition and mixing problems. Similar results have been reported by other researchers [12,15,16]. Enzymatic hydrolysis of various lignocellulosic materials using a variety of enzyme sources has been reported by other researchers. In a study, a comparison was made between the use of celluclast and a combination of celluclast and *Periconia* sp. bcc 2871 as the source of enzyme for hydrolysis of rice straw and a reducing sugar production of 84 and 132 mg/g substrate was reported, respectively [17]. Cellulase from *T. reesei* ZM4F3 was used for enzymatic hydrolysis of rice straw and as a result, 733 mg/g substrate reducing sugar was produced [17]. Use of cellulase by *Trametes hirsuta* for enzymatic hydrolysis of rice straw resulted in the production of 685 mg/g substrate reducing sugars and a hydrolysis yield of 88% was achieved [1]. Rice straw was enzymatically hydrolyzed by cellulases from *T. reesei* A1 and *Penicillium* sp. B1. As a result, a reducing sugar concentration of 374 and 316 mg/g substrate and a hydrolysis yield of 70.50% and 57.90% were obtained, respectively [18]. As reported in a research, enzymatic hydrolysis of rice hull by a combination of celluclast 1.5 L and Novozyme 188 resulted in the production of 154 mg/g substrate reducing sugar and 32% hydrolysis yield was achieved [19]. Bagasse was hydrolyzed by cellulases from *Penicillium janthinellum* NCIM 1171 and 846 mg/g substrate reducing sugar equivalent to 94.60% hydrolysis yield was obtained [20]. Enzymatic hydrolysis of maize straw by cellulase from *T. reesei* ZU-02 produced 814 mg/g substrate reducing sugar and 83.5% conversion yield was obtained [14]. In this study, as a result of enzymatic hydrolysis of alkali-pretreated rice straw using cellulase from *T. harzianum* SNRS3, a reducing sugar yield of 600 mg/g substrate and a hydrolysis yield of 60.75% were achieved, as compared to reducing sugar production of 800 mg/g substrate and 81.35% conversion yield obtained when celluclast was used.

As a result of enzymatic hydrolysis of alkali-pretreated rice straw using cellulase from *T. harzianum* SNRS3, a reducing sugar yield of 600 mg/g substrate and a hydrolysis yield of 60.75% were achieved.

### FPase as the rate-limiting enzyme

Cellulose enzymatic hydrolysis needs all the three componets of cellulase enzyme complex system to act synergistically. However, to study the significant role of FPase in cellulose enzymatic hydrolysis, four combinations of cellulase enzyme were used in this study. The reducing sugar profile was studied over a period of 96 h. Table 6 shows that the production of reducing sugar was increased significantly (*p* < 0.05) according to the concentration of FPase. As for the effect of incubation time, the reducing sugar production was significantly increased (*p* < 0.05) from 0-72 h for all the enzyme concentrations used in enzymatic hydrolysis of rice straw. The detail of the statistical analysis is presented in Table 7.

**Table 6:** **Reducing sugar production using celluclast and crude cellulase from** ***T. harzianum*** **SNRS3 at different concentrations of FPase (U/mL) and β-glucosidase (U/ mL)**

**Enzyme source**	**FPase**	**β-glucosidase**	**Reducing sugar**	**Glucose**
	**(U/mL)**	**(U/mL)**	**(g/L)**	**(g/L)**
Celluclast (5 FPU)	9.90 ± 0.07	5.55 ± 0.88	36.30 ± 0.26	11.00 ± 0.45
Crude cellulase	9.86 ± 0.05	265.20 ± 7.22	26.33 ± 0.75	10.02 ± 0.2
from *T. harzianum*				
SNRS3				
Celluclast (20 FPU)	39.60 ± 0.08	22.20 ± 0.84	57.27 ± 0.63	21.15 ± 0.89
Crude cellulase	0.83 ± 0.07	22.10 ± 0.33	5.94 ± 0.31	1.16 ± 0.1
from *T. harzianum*				
SNRS3				

Note: Values are means of 3 replicates ± SD.

**Table 7:** **Reducing sugar production from rice straw using celluclast and crude cellulase from** ***T. harzianum*** **SNRS3 at different concentrations of FPase (U/mL) and β-glucosidase (U/ mL)**

**Time (h)**	**Enzyme Concentration (U/mL)**			
	*1	*2	*3	*4
0	0.00^Aa^	0.00^Aa^	0.00^Aa^	0.00^Aa^
24	23.55^Ba^	16.15^Bb^	39.56^Bc^	4.47^Bd^
48	29.19^Ca^	22.28^Cb^	51.10^Cc^	5.05^Cd^
72	36.30^Da^	26.33^Db^	57.27^Dc^	5.94^Dd^
96	37.36^Da^	27.56^Db^	58.89^Dc^	6.23^Dd^

Note: *1: Celluclast: FPase 9.90 U/mL, β-glucosidase 5.55 U/mL.

*2: Crude cellulase: FPase 9.86 U/mL, β-glucosidase 265.20 U/mL.

*3: Celluclast: FPase 39.60 U/mL, β-glucosidase 22.20 U/mL.

*4: Crude cellulase: FPase 0.83 U/mL, β-glucosidase 22.10 U/mL.

Means expressed with different superscript capital letters within the same column was significantly different at *p* < 0.05.

Means expressed with different superscript small letters within the same row was significantly different at *p* < 0.05.

As shown in Table 6, at 72 h of enzymatic hydrolysis and at concentration of FPase, 9.9 U/mL and 9.86 U/mL for celluclast and the crude cellulase, respectively, regardless of the significant difference in the concentration of β-glucosidase present in celluclast (5.55 U/mL) and that in the crude cellulase (265.2 U/mL), 36.30 g/L and 26.33 g/L reducing sugars were obtained by using celluclast and the crude cellulase of *T. harzianum* SNRS3, respectively. Moreover, glucose concentration was detected at 11.0 g/L and 10.02 g/L for celluclast and the crude cellulase, respectively. However, when β-glucosidase activity was fixed at 22.2 U/mL and 22.1 U/mL for celluclast and crude cellulase while FPase concentration for celluclast and the crude cellulase was different at the activity of 39.6 U/mL and 0.83 U/mL for celluclast and the crude cellulase, respectively, the reducing sugar analysis revealed a much higher production of reducing sugars by celluclast, as compared to the crude cellulase. As a result, 57.27 g/L and 5.94 g/L reducing sugars were obtained by using celluclast and the crude cellulase, respectively. The results of HPLC analysis revealed that glucose at the concentration of 21.15 g/L and 1.16 g/L was obtained when celluclast and the crude cellulase were used, respectively.

Cellulases consist of three main enzymes, endoglucanase or CMCase, exoglucanase or cellobiohydrolase and β-glucosidase, which act synergistically in the cellulose hydrolysis process [21-26]. Endoglucanase attacks intramolecular β-1,4-glucosidic bonds in cellulose polymeric chain, leaving new chain ends for exoglucanase to cleave. Exoglucanase or cellobiohydrolase is the second hydrolytic enzyme attacking cellulose chain in the ends producing cellobiose or glucose, while β-glucosidase converts cellobiose to glucose. Therefore, endoglucanase and exoglucanase are mainly responsible for depolymerization of cellulose polymer chains and this enzymatic depolymerization step is the rate-limiting step in cellulose hydrolysis process [27]. The effect of different ratios of cellulase and β-glucosidase on glucose production was studied and it was shown that the ratio of cellulase to β-glucosidase 5:1 gave higher glucose concentration, as compared to the ratio of 1: 5, where the lowest glucose concentration was produced. In fact, the latter ratio only resulted in the excess of β- glucosidase in the reaction mixture [3]. Therefore, the results of this study are in accordance with those reported in other research [3,27] that laid emphasis on the need for the presence of FPase at a substantial concentration in the cellulase enzyme complex for an efficient hydrolysis. From the results of this study, it is inferred that for an efficient cellulose enzymatic hydrolysis, the presence of the whole set of cellulose hydrolytic enzymes is required. FPase is a major component of cellulase enzymes complex, lack of which causes inefficient cellulose hydrolysis. This leads to a low production of reducing sugars and the presence of β-glucosidase at a high concentration would not help in such a condition since this latter enzyme is only accumulated as the relative activity of β-glucosidase to FPase is much higher.

### Production of ABE by *C. acetobutylicum* ATCC 824 using rice straw hydrolysate

**Figure 4:**
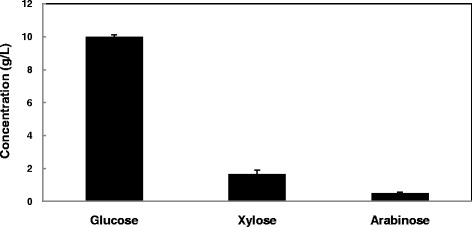
**Composition of rice straw hydrolysate.** Values are means of 3 replicates ± SD.

Rice straw hydrolysate was used as the substrate for ABE production. Based on the results of HPLC, rice straw hydrolysate contained glucose, xylose, and arabinose as individual sugars. Concentration of the individual sugars is shown in Figure 4.

**Figure 5:**
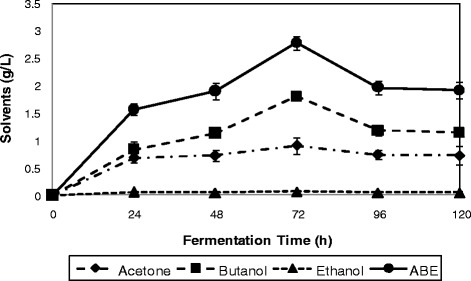
**Production of ABE from glucose by** ***C. acetobutylicum*** **ATCC 824.** Values are means of 3 replicates ± SD. Symbols represent:  Acetone  Butanol  Ethanol  ABE.

**Figure 6:**
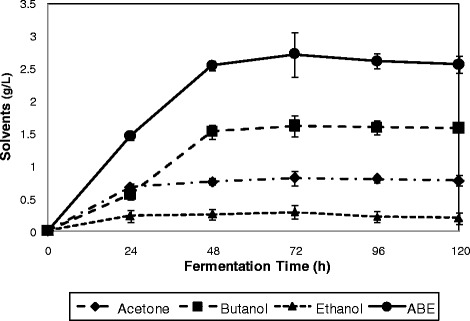
**Production of ABE from rice straw hydrolysate by** ***C. acetobutylicum*** **ATCC 824.** Values are means of 3 replicates ± SD. Symbols represent:  Acetone  Butanol  Ethanol  ABE.

In order to investigate the application of fermentable sugars produced from pretreated rice straw using the crude cellulase, rice straw hydrolysate obtained, was employed as fermentation substrate in ABE fermentation by *C. acetobutylicum* ATCC 824. In order to compare the results of ABE fermentation obtained in this study, glucose (10 g/L) was used as a model substrate. As can be seen from Figure 5, fermentation of (10 g/L) glucose as a model substrate resulted in the production of a total ABE concentration of 2.79 g/L (0.91 g/L acetone, 1.81 g/L butanol, and 0.07 g/L ethanol). While, as illustrated in Figure 6, the results of rice straw hydrolysate fermentation by *C. acetobutylicum* ATCC 824 indicates a maximum total ABE production of 2.73 g/L (0.82 g/L acetone, 1.62 g/L butanol, and 0.29 g/L ethanol).

In a research, fermentation of 10 g/L, and 20 g/L glucose by *C. acetobutylicum* ATCC 824, resulted in the production of 1.76 g/L total ABE (0.41 g/L acetone, 1.18 g/L butanol, and 0.17 g/L ethanol), and 5.76 g/L total ABE (1.36 g/L acetone, 4.08 g/L butanol, and 0.32 g/L ethanol), when 10 g/L, and 20 g/L glucose were used, respectively [28]. In another study, domestic organic waste containing (14 g/L glucose) as carbon source was used in fermentation by *C. acetobutylicum* DSM 1731 and 1.5 g/L total ABE was produced [29].

In fermentation of rice straw hydrolysate, at 48 h of fermentation, glucose as the primary and preferred carbon source was totally consumed. It is worth noticing that Clostridia are able to consume pentoses as well as hexoses [30]. However, arabinose was not consumed by the strain, whereas xylose was consumed. Hence, fermentable sugars produced via enzymatic hydrolysis of rice straw showed to be potential sugars as substrate for ABE production and can be applied for production of biofuels such as biobutanol.

The results of kinetic studies are indicated in Table 8. As can be seen from the table, kinetic studies indicated a cell yield and biomass productivity of 0.19 g cell/g glucose, and 0.04 g/L/h, respectively. Fermentation of rice straw hydrolysate resulted in an ABE yield and productivity of 0.27 g/g glucose, and 0.04 g/L/h, respectively. A butanol yield of 0.16 g/g glucose was achieved.

**Table 8:** **Performance of ABE fermentation by** ***C. acetobutylicum*** **ATCC 824 on rice straw hydrolysate**

**Parameter**	**ABE fermentation by** ***C.acetobutylicum*** **ATCC 824**
Initial glucose concentration (g/L)	10
Final glucose concentration (g/L)	0
Maximum acetone concentration(g/L)	0.82 ± 0.10
Maximum butanol concentration(g/L)	1.62 ± 0.16
Maximum ethanol concentration(g/L)	0.29 ± 0.12
Total solvents concentration(g/L)	2.73 ± 0.34
Fermentation time(h)	72
Solvents yield(g ABE/g glucose)	0.27
Butanol yield (g butanol/g glucose)	0.16
Yield of cells (g cells/g glucose)	0.19
Solvents productivity (g/L/h)	0.04
Productivity of biomass (g/L/h)	0.04

## Conclusions

The present study confirmed that the use of NaOH as an alkali reagent for pretreatment of rice straw could be effective. The subsequent enzymatic hydrolysis of alkali-pretreated rice straw and the use of rice straw hydrolysate as the substrate for biobutanol production was shown to be successful. The use of 2% (w/v) NaOH was shown to be the most effective concentration of NaOH for pretreatment of rice straw. Enzymatic hydrolysis of rice straw pretreated with 2% (w/v) NaOH resulted in a reducing sugar yield of 0.6 g/g substrate and 60.75% saccharification was obtained. Reducing sugar production increased with the increase in the concentration of the enzyme and a similar trend was observed between fermentable sugar production and hydrolysis yield (%).With the rise in substrate concentration, fermentable sugar production increased as well. However, an opposite trend was observed between substrate concentration and hydrolysis yield (%) due to stirring difficulties, poor mixing, and end product inhibition. The use of 5% (w/v) substrate resulted in the highest concentration of reducing sugars. Rice straw hydrolysate was successfully utilized as substrate for biobutanol production resulting in an ABE yield and productivity of 0.27 g/g glucose and 0.04 g/L/h, respectively. A butanol yield of 0.16 g/g glucose was achieved. The study also confirmed that, as an enzyme complex, in order for cellulase to be efficient, the presence of all the components at substantial concentration is highly required and that the presence of β-glucosidase at a high concentration would not help in case FPase is not present at high enough concentration to initiate the cellulolytic reaction. In fact, in such a condition the addition of β-glucosidase is not effective since this enzyme is only accumulated due to the lack of cellobiose, the substrate for this enzyme.

## Methods

### Preparation and pretreatment of the substrate

Rice straw was collected from a paddy field in Sekinchan, Selangor, Malaysia. By using an electric grinder (Model CW-1, Hsiang Tai, Taiwan, 220 V, 50 Hz, 10 A), rice straw was ground to 2 mm length in size, kept in a cold room at 4°C prior to use. Pretreatment method was described in detail in our previous paper [13]. Rice straw pretreatment was performed by using different concentrations of NaOH [1%, 2%, 3%, and 4% (w/v)]. Effect of NaOH at various concentrations on substrate pretreatment and the subsequent sugar production was investigated.

### Microorganism and inoculum preparation

A local isolate of *T. harzianum* SNRS3 was used in this study. The fungus was isolated from rice straw obtained from a rice field in Sekinchan, Selangor, Malaysia (unpublished data). Inoculum preparation was described in our previous study [13].

### Solid state fermentation of rice straw by *T. harzianum* SNRS3 for cellulase production

Solid state cultivation was conducted and the crude enzyme mixture was extracted as described in the previous research [13]. The crude enzyme was kept at 4°C prior to use for enzymatic hydrolysis.

### Enzymatic hydrolysis

#### Effect of pretreatment of rice straw using NaOH at various concentrations on reducing sugar production

To investigate the most effective concentration of NaOH for pretreatment of rice straw as the substrate for saccharification, NaOH at different concentrations [1%, 2%, 3%, and 4% (w/v)] was used. Untreated substrate was used as control. Pretreated substrate was placed in 50 mL Erlenmeyer flasks followed by being autoclaved at 121°C, for 15 min. Crude cellulase enzyme was added to each flask at the concentration of FPase 6.25 U/g substrate, CMCase 111.31 U/g substrate and β-glucosidase 173.71 U/g substrate. Enzymatic hydrolysis of alkali-pretreated rice straw was carried out in a reaction mixture containing [5% (w/v)] substrate in 20 mL 50 mM sodium citrate buffer (pH 5). The reaction mixture was incubated in a shaker incubator at 50°C and rotated at 150 rpm for 96 h. Samples (1 mL) were withdrawn at 0, 24, 48, 72 and 96 h. Samples were centrifuged for 10 min at 10,000 rpm. The supernatant was used for reducing sugar analysis and kept at -20°C prior to analysis. Concentration of NaOH yielding the highest concentration of reducing sugars was applied for pretreatment of rice straw in subsequent study.

#### Effect of enzyme loadings on reducing sugar production

Effect of enzyme loadings on enzymatic hydrolysis was investigated using rice straw pretreated with 2% (w/v) NaOH as the substrate. In order to study the effect of enzyme concentration on sugar production, preparation of the flasks and the substrate was done as described above. [5% (w/v)] substrate pretreated with 2% (w/v) NaOH was placed in each flask autoclaved at 121°C, for 15 min. It was then followed by the addition of various concentrations of the enzyme, each in a separate flask. Enzymatic saccharification was performed under the same conditions as described above and the samples were taken at regular interval. Samples were centrifuged and kept at -20°C prior to use for sugar analysis. Total activity of the enzyme was calculated based on the following equation:

1 $$ Total\  activity\  of\  the\  enzyme\left(U/g\right)= Enzyme\  activity\ \left(U/ mL\right)x\  Volume\  of\  the\  enzyme(mL)x\ {10}^a $$

^a^coefficient of the conversion of unit of enzyme activity (U/ mL) to (U/g substrate).

Enzyme activity was calculated based on the following Equations:

2 $$ FPase\ \left(U/ mL\right)=\frac{final\  abs-c}{m}\ x\frac{df}{sample\  volume}x\frac{1}{time}x\frac{1000\ \mu g}{1\  mg}x\frac{1\ \mu mole}{180.16\ \mu g} $$

3 $$ CMCase\ \left(U/ mL\right)=\frac{final\  abs-c}{m}\ x\frac{df}{sample\  volume}x\frac{1}{time}x\frac{1000\ \mu g}{1\  mg}x\frac{1\ \mu mole}{180.16\ \mu g} $$

4 $$ \beta - glucosidase\ \left(U/ mL\right)=\frac{final\  abs-c}{m}\ x\frac{df}{sample\  volume}x\frac{1}{time}x\frac{1000\ \mu g}{1\  mg}x\frac{1\ \mu mole}{139.1\ \mu g} $$

Where m is the slope from the standard curve, abs is the absorbance, c is the intercept, and df is the dilution factor. Time is the reaction time and is expressed in minute. To convert U/mL to U/g, the value for the activity of the enzyme (U/mL) was divided by the amount of rice straw in each flask (3 g) times volume of sodium citrate buffer used to extract the enzyme (30/mL).

#### Effect of substrate concentration on reducing sugar production

To investigate the effect of substrate concentration on enzymatic hydrolysis [1%, 3%, 5%, and 7% (w/v)] substrate, pretreated with 2% (w/v) NaOH was placed in different flasks. Crude cellulase concentration yielding the highest reducing sugar concentration from the previous study determined the enzyme concentration to be added to the substrate in each flask. Therefore, crude cellulase at the concentration of FPase 93.75 U/g substrate, CMCase 1669.65 U/g substrate and β-glucosidase 2605.65 U/g substrate was added to the substrate in flasks, each containing a different concentration of the substrate. Sampling was done at regular interval and the samples were centrifuged at 10,000 rpm for 10 min followed by being kept at -20°C. All the experiments were done in triplicates.

### FPase as the rate-limiting enzyme

To study the significant role of FPase in cellulose enzymatic hydrolysis, FPase activity was fixed both for celluclast (5 FPU) and the crude cellulase from *T. harzianum* SNRS3. FPase activity was fixed at 9.90 U/mL and 9.86 U/mL for celluclast and the crude cellulase, respectively. However, β-glucosidase activity for celluclast and the crude cellulase enzyme was significantly different at 5.55 U/mL and 265.2 U/mL, respectively. Enzymatic hydrolysis of 5% (w/v) rice straw was carried out in 20 mL of 50 mM sodium citrate buffer solution, using a shaker incubator at 50°C, 150 rpm for 96 h. The initial pH was adjusted to 5.0 which is the optimum pH for celluclast [31]. Alternatively, in another experiment, β-glucosidase activity was fixed both for celluclast (20 FPU) and the crude cellulase enzyme. β-glucosidase activity was fixed at 22.20 U/mL and 22.10 U/mL for celluclast and the crude cellulase enzyme, respectively. However, the concentration of FPase in celluclast and the crude cellulase enzyme was significantly different at 39.6 U/mL and 0.83 U/mL, respectively. The enzymatic hydrolysis was performed under the afore-mentioned conditions. Samples were kept at -20°C prior to the analysis of reducing sugar concentration.

### Substrate preparation for Acetone-Butanol-Ethanol (ABE) fermentation

As substrate for ABE fermentation, rice straw pretreated with 2% (w/v) NaOH was used. Following pretreatment, the pretreated substrate was subjected to enzymatic hydrolysis as described earlier and the enzyme and substrate concentration were used according to the results of the previous experiments. Rice straw hydrolysate was used as the substrate in ABE fermentation for the growth of the bacteria and subsequent biobutanol production.

### Inoculum maintenance and preparation for ABE fermentation

*C. acetobutylicum* ATCC 824 from American Type Culture Collection was used as the ABE producing microorganism. The stock culture was grown on Reinforced Clostridial Medium (RCM) agar under anaerobic conditions using anaerobe container system Gaspak^TM^ EZ at 37°C. The single colony was grown on RCM broth at 37°C for 48 h. The medium was sparged with N_2_ gas to provide anaerobic conditions for the growth of the bacteria. Bacterial cells were maintained as spore suspension on 60% (v/v) sterile glycerol at the ratio 2: 1 (culture: glycerol) and stored at -40°C prior to use.

Chemical composition and RCM preparation method is as follows:

- RCM is composed of Meat extract 10 g/L, Peptone 5 g/L, Yeast extract 3 g/L, D-(+)-glucose 5 g/L, Starch 1 g/L, NaCl 5 g/L, Sodium acetate 3 g/L, L-Cysteine hydrochloride 0.5 g/L, Agar 0.5 g/L. Medium pH was adjusted to pH 6.0 using 1 M sodium hydroxide (NaOH) before being transferred into serum bottles. 100 mL of the prepared media was transferred into 125 mL serum bottles and sparged with nitrogen gas for 15 min and closed tightly using rubber septa before it was autoclaved at 121°C for 15 min. The media was prepared a day before inoculum preparation and stored at 4°C prior to use for inoculum preparation. Inoculum was heat shocked at 80°C for 2 min prior to being inoculated into freshly prepared RCM. Inoculum was then kept at 37°C for 48 h before being transferred into fermentation production medium.

### ABE production medium

Tryptone-Yeast extract-Acetate (TYA) medium was used as ABE production medium. Chemical composition and preparation method is as follows:

Yeast extract 2 g/L, Tryptone 6 g/L, CH_3_COONH_4_ 3 g/L, FeSO_4_.7 H_2_O 10 mg/L, KH_2_PO_4_ 0.5 g/L, MgSO_4_ 0.3 g/L, rice straw hydrolysate (10 g/L glucose, 1.66 g/L xylose, 0.5 g/L arabinose). For preparation of TYA medium, all the chemical components were dissolved in rice straw hydrolysate (carbon source) and transferred into 125 mL serum bottles. Medium pH was adjusted to 5.5. The medium was then sparged with nitrogen gas for 15 min for anaerobiosis before being autoclaved at 115°C for 10 min. 5% of freshly prepared inoculum was inoculated into fermentation production medium. Fermentation was carried out at 37°C, 120 rpm for 120 h. Sampling was done at 0, 24, 48, 72, 96, and 120 h. Samples were kept at -20°C prior to being analyzed for solvents and optical density.

### Analytical procedure

Cellulose, hemicelluloses, lignin, and ash content of rice straw were measured using a standard method [32]. Reducing sugar analysis was performed by using Dinitrosalicylic acid (DNS) reagent and according to a standard method [33]. Enzyme activity was assayed according to the standard method [34]. (%) Saccharification was calculated according to the following Equation: (%) Saccharification = reducing sugars × 0.9 × 100)/ carbohydrates in substrate [1]. Bacterial cell concentration was determined based on correlation between dry cell weight (DCW) and optical density (OD) using spectrophotometer at 680 nm. Individual sugars concentration was measured by using High Performance Liquid Chromatography (HPLC). Solvents were analyzed by using Gas Chromatography (GC). Yield was defined as grams of ABE produced per gram of glucose utilized. ABE productivity was calculated as total ABE produced in g/L divided by the fermentation time and is expressed as g/L/h.

Yield of cells was defined as grams of cells produced per grams of glucose utilized. Productivity of biomass was calculated as maximum cell concentration in g/L divided by the fermentation time and is expressed as g/L/h.

### Statistical analysis

The data was analyzed by one-way analysis of variance (ANOVA). t Tests (LSD) was used to compare the difference of means among treatment groups. Differences of *p* < 0.05 were considered significant.
